# *Mycobacterium bovis* vaccination and subsequent experimental infection outcomes are associated with changes in vitamin D status in dairy calves

**DOI:** 10.3168/jdsc.2024-0547

**Published:** 2024-05-10

**Authors:** Rachel L. Lee, Kieran G. Meade, Shelley G. Rhodes, Tom Ford, Ilias Kyriazakis

**Affiliations:** 1 Institute for Global Food Security, School of Biological Sciences, Queen's University, Belfast, United Kingdom BT9 5DL; 2 School of Agriculture and Food Science, University College Dublin, Belfield, Dublin 4, Ireland D04 C1P1; 3 Department of Bacteriology, Animal and Plant Health Agency, New Haw, Addlestone, Surrey, United Kingdom KT15 3NB; 4 Veterinary Sciences Division, Agri-Food and Biosciences Institute, Stormont, Belfast, Northern Ireland BT9 5PX

## Abstract

Investigations into the role of vitamin D (vitD) in the immune response of cattle are limited. The objectives of this study were therefore to investigate the association between circulating vitD concentration, tuberculosis (TB) vaccination, and *Mycobacterium bovis* infection outcomes in 24 dairy calves (<8 wk old) that were housed throughout and fed a BW-based allowance. The study design incorporated 2 phases: vaccination (experimental wk 0–52) and experimental infection phase (wk 52–65). Vaccinated calves (n = 12) received a subcutaneous injection of a live attenuated TB strain at wk 0, whereas unvaccinated ones (n = 12) were injected with saline. All animals were infected with 7,600 cfu of *M. bovis* 52 wk postvaccination, and lung and lymph node tissues were assessed for pathology following euthanasia after wk 65. Blood samples were taken throughout wk 0 to 65. Serum 25-hydroxyvitamin D [25(OH)D] concentrations were measured using a commercial ELISA. A mixed-effects linear regression model revealed significantly higher 25(OH)D concentrations in the vaccinates postinfection (wk 65) compared with the nonvaccinates. Linear regression analysis between 25(OH)D concentration and the level of *M. bovis*-driven pathology revealed a negative linear relationship (i.e., higher concentrations were associated with lower pathology scores) irrespective of vaccination status. No correlation was detected between IFN-γ cytokine production and vitD concentration. Overall, the results support an impactful role for vitD in the development of effective immunity of cattle against *M. bovis*. Gaining insight into the interaction between TB vaccination, *M. bovis* infection, and vitD could potentially guide the optimization of vaccination protocols and future TB control strategies.

## Highlights

• Bacille Calmette-Guerin (BCG) vaccination does not affect vitD concentrations pre-infection.
• Mycobacterium bovis infection may cause pathogen-induced anorexia in cattle.
• Vitamin D concentrations differ postinfection depending on vaccination status.
• Higher levels of vitD are associated with lower tuberculosis pathology scores.
• Vitamin D levels are not associated with the production of cytokine interferon-γ.

## Graphical Abstract

**Figure:**
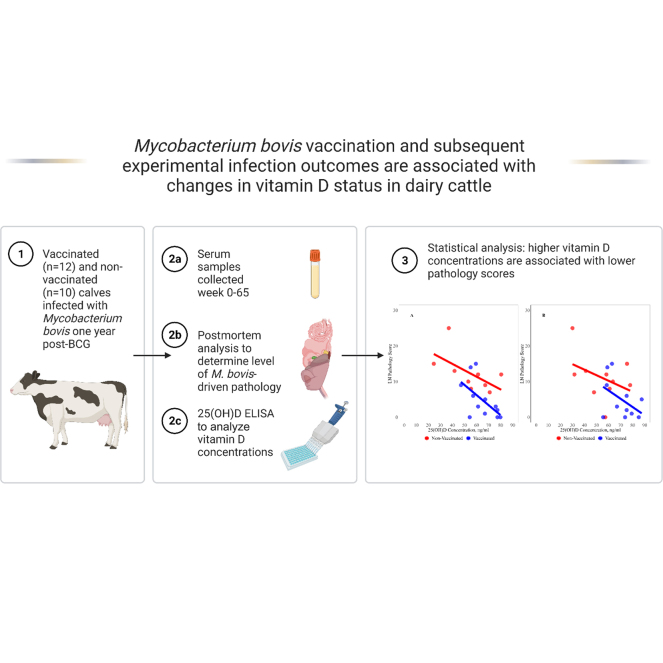
**Summary:** The study design incorporated 2 phases: the vaccination phase (weeks 0-52) and the experimental infection phase (weeks 52-65). Twenty-eight cattle younger than 8 weeks old were housed at APHA Weybridge throughout the study and fed a body weight-controlled diet. Twenty-two calves in total were included for statistical analyses. A mixed-effects logistic regression model was constructed using the nlme package within R, revealing that vitamin D (vitD) concentrations increased in vaccinates compared with nonvaccinates postinfection, with vaccinated animals reaching higher vitD levels than nonvaccinates by week 65. Linear regression analysis revealed a negative linear relationship between vitD concentration and pathology score irrespective of vaccination status. There was no correlation between interferon-γ cytokine production and vitD concentration. Results suggest that vitD may play a role in the reduction of pathology of bovine tuberculosis. Created with BioRender.com.

Vitamin D (**VitD**) has an established role in the regulation of calcium-phosphate homeostasis and skeletal health. However, vitD receptor and activating enzyme 1α-hydroxylase expression have been identified in several cells involved in immune system functions and therefore the role of vitD on immune related functions is of increasing interest (D'Amelio et al., 2012). The optimal levels of circulating 25-hydroxyvitamin D [**25(OH)D**] for immune function are not well defined; however, concentrations below 30 ng/mL have been shown to have a negative effect on immune cell profiles in cattle (Flores-Villalva et al., 2021).

Specifically in relation to mycobacteria, there is evidence for vitD regulation of antimycobacterial responses (e.g., by inhibiting intracellular replication, promoting the maturation and activation of monocytes and macrophages, and the induction of nitric oxide synthase) (Rode et al., 2017). Lower concentrations of 25(OH)D have been found in human patients with active tuberculosis (**TB**) in comparison to healthy patients; however, it is unclear if this difference is caused by the infection or if disease progression is favored by lower 25(OH)D levels (Huang et al., 2017). Several in vitro human medicine studies support an anti-inflammatory role of vitD, without a reduction in antimycobacterial activity, which likely reduces the tissue damage characteristic of TB immunopathology (Torres-Juarez et al., 2015; Papagni et al., 2022).

Vaccination with Bacille Calmette-Guerin (**BCG**), a live attenuated vaccine form of *Mycobacterium bovis*, represents a potentially viable control measure for reducing bovine tuberculosis (**bTB**) prevalence in cattle (Milián-Suazo et al., 2022). Studies of BCG vaccination have shown substantial protection against both experimental and natural infection with *M. bovis* and a recent meta-analysis of the efficacy of BCG supports its use (Srinivasan et al., 2021). Studies in the human medicine literature report interactions between 25(OH)D concentrations and vaccination responses, influencing the hypotheses for our study that an interaction between BCG vaccination and *M. bovis* infection could influence 25(OH)D concentrations (Shahini et al., 2023).

The aims of this study were to investigate the effects of vaccination and subsequent infection with *M. bovis* on the circulating levels of 25(OH)D in dairy cattle, and to then compare these levels of 25(OH)D concentration with pathology in vaccinated and unvaccinated cattle. As IFN-γ is both a major protective cytokine for TB and a potent inflammatory mediator, we also investigated whether there was an association of IFN-γ with 25(OH)D (Kumar, 2017). The 3 main hypotheses were (1) BCG vaccination alone would not affect circulating 25(OH)D levels; (2) there would be differences in circulating 25(OH)D levels between vaccinated and unvaccinated cattle postinfection (**pi**); and (3) vaccination and 25(OH)D concentrations would both play a role in the reduction of TB pathology.

All procedures conducted were approved by the Animal Plant and Health Agency (**APHA**) Animal Welfare and Ethical Review Board in accordance with the Animal Scientific Procedures Act (ASPA, 1986) at APHA (PFD840A5–2-006V2). As a result of existing approval, ethical exemption was provided by the Animal Research Ethics Committee, UCD (AREC-E-22–34-Meade).

Twenty-eight castrated male Danish Holstein or Holstein-crossbred calves were sourced from Denmark (an officially bovine TB-free country) and housed at APHA Weybridge (51°N) throughout the experiment. Calves were 31 to 46 d old on arrival at APHA in July 2022 (Supplemental Table S1; see Notes).

The initial sample size was based on a power analysis with a 5% significance level and 95% power, considering the reduction of pathology as the primary endpoint. Calculated from a median pulmonary lymph node score of 16 (control) and 5 (BCG; Jones et al., 2022) with an overall SD of 6.6, the target size was 10 animals per group. This was adjusted to 12 animals per group to account for potential losses due to infection.

Calves were raised for 4 wk on Vita Milk Omega Gold milk replacer (containing 5,000 IU of vitamin D_3_/kg) and Heygates Course Calf Mixture feed (2,200 IU of vitamin D_3_/kg) and switched to ForFarmers Grower Pellet (2,500 IU of vitamin D_3_/kg) for the remainder of the study. They were fed a BW-based allowance and had access to ad libitum grass hay and straw.

The study consisted of 2 phases: vaccination (wk 0–52) and experimental infection (wk 52–65). Animals were randomly allocated to treatment groups and balanced by breed. For the vaccination phase, the vaccination group (n = 14) received a 0.5-mL subcutaneous injection of a live attenuated TB strain (BCG Danish strain 1331; AJVaccines, Copenhagen, Denmark) 2 wk after arrival (wk 0 of the study). The unvaccinated group (n = 14) received a 0.5-mL subcutaneous injection of PBS. The groups were kept separately within the barn to link observed effects more confidently to the vaccination, such as potential effects on feed intake. Natural light within the building was not controlled and artificial lights were manually switched on and off by technicians. Pens inside the barn were equidistant from window light sources.

Before *M. bovis* challenge on wk 52, animals were moved to a CL3 containment unit; animals were randomly assigned to the 6 animal rooms by staff so that 2 vaccinates and 2 nonvaccinates were housed together. Two animals were randomly deselected for infection due to the capacity of the CL3 unit. Another 2 animals were lost to the study due to study-unrelated causes pre-infection, leaving a total of 24 animals within the study (12 per group). Both groups were experimentally infected via the endotracheal/endobronchial route with virulent strain AF2122/97 of *M. bovis* in wk 52 (Jones et al., 2022). The CL3 facility was under negative pressure, with each pen having a separate high-efficiency particulate air-filtered duct from each room. The light within the containment facility was scheduled.

Blood samples (per animal; one clotted sample tube for serum collection and one heparin-coated sample tube for IFN-γ) were collected on experimental wk 0 (pre-BCG), 4, 6, 8, 16, 24, 32, 40, 45, 47, 50, 53 (1 wk pi), 54, 58, 62, and 65. Body weight measurements were obtained at all sampling points before the infection (wk 0–52) using calibrated scales. All animals were killed 3 to 5 d after wk 65 samples were taken, using an appropriate method under the ASPA (1986) Schedule 1, consisting of captive bolt stunning and exsanguination.

Serum samples were analyzed for concentrations of total 25(OH)D using a commercially available ELISA (VID3-K01, Eagle BioScience, Nashua, NH) with customized bovine standards (Nelson et al., 2016). The concentrations of 25(OH)D were determined by measuring the absorbance at a wavelength of 450 nm with a reference wavelength of 620 nm using a CLARIOstar Plus BMG LabTech microplate reader. The resulting absorbance values were fitted against the bovine standard concentrations to a standard curve using cubic spine plotting in GraphPad Prism v9 software to convert to 25(OH)D concentrations. Standards were run in duplicate, and samples were run singly. The intra-assay CV was 1.41% based on measurements of replicates of the bovine standards within a single assay. The inter-assay CV was 3.60%, determined from 9 independent assays performed on separate days. Interferon-γ was assessed using BOVIGAM ELISA provided by APHA (Holder et al., 2023).

Postmortem examinations were conducted 3 to 5 d following the last blood sampling week (wk 65) to determine the level of pathology observed in the lungs and lymph nodes (**LN**) using a previously published method and scoring system (Lyashchenko et al., 2004; Jones et al., 2022). Briefly, the LN (submandibular, medial retropharyngeal, mediastinal, bronchial, tracheobronchial) and lung lobes were meticulously examined postmortem. Individual LN and lung scores were added up to calculate the total scores.

All statistical analyses were conducted in R (version 4.2.3), including but not limited to packages *nlme, lme,* and *anova* (Pinheiro and Bates, 2000). Two unvaccinated calves were excluded from the analysis due to missing data points throughout wk 0 to 65; therefore, the final analysis included 12 vaccinated and 10 unvaccinated calves.

The effect of vaccination status on BW and 25(OH)D concentrations was analyzed using linear mixed effects (**LME**) models. The main fixed effects in both models were date of breed, vaccination status, and time; BW at allocation (**BW0**) was a covariate. Calf ID was the random effect, as it formed the repeated measure in the dataset. Body weight or 25(OH)D concentrations were the dependent variable. The normality of residuals was assessed using QQ plots and the Shapiro-Wilk test, concluding normal distribution of the residuals. Heteroscedasticity was evaluated through visual inspection of residual plots associated with the LME model. The absence of discernible patterns or trends suggested that the assumption of homogeneity of variance was met across the observed data points. Boxplots of BW and 25(OH)D concentrations against time were plotted to determine statistical outliers (Supplemental Figure S1, see Notes). The screening of interaction terms assessed their impact by systematically evaluating significance with the Akaike information criterion (**AIC**). Noncontributing terms were then identified and removed (Supplemental Table S2, see Notes). The choice of covariance structures in the mixed-effects model was determined through systematic evaluation based on AIC and Bayesian information criterion (Supplemental Tables S3 and S4, see Notes). Two structures (spatial power and ante-dependence) were considered due to the uneven spacing of the sampling points. The ante-dependence structure was selected for its lowest AIC value, indicating superior fit. Significance was determined at *P* < 0.05 and tendency was declared at 0.05 ≤ *P* < 0.10. Post hoc analysis was conducted to identify where significant interactions occurred, and estimated marginal means were obtained. Pairwise comparisons were performed using Tukey's honestly significant difference (HSD) multiple comparison test on the interaction between vaccination status and time.

An investigation into the relationship between the independent variables, vaccination status, and 25(OH)D concentration and the dependent variable pathology score used separate linear regression models for each time point pi, using the *lm* function in R. Before model fitting, assumptions of normality, linearity, and homoscedasticity were assessed as above. To confirm the linearity assumption, scatter plots of the predictor variables against the fitted values and residuals were examined. Focused on time points where 25(OH)D concentrations tended to or significantly influenced pathology scores (wk 62 and 65), Pearson correlation coefficients (ρ) were computed to quantify the linear relationship. Finally, the relationship between 25(OH)D concentrations and IFN-γ was analyzed through a partial correlation analysis. The analysis controlled for the effects of vaccination status and time. The partial correlation coefficient (r) was computed using the *pcor* function.

A main effect of time on BW was found pre-infection, with animals increasing in BW as time progressed (*F* = 670; *P* < 0.001). Main effects of vaccination status and breed on BW were not significant, nor were the effects of their interactions.

An interaction between vaccination status and time on 25(OH)D concentrations was observed [*F*(15, 298) = 2.67; *P* = 0.0008]. There was no effect of vaccination on 25(OH)D concentration before infection, but there were higher 25(OH)D concentrations in the vaccinated calves compared with the unvaccinated ones pi (wk 65: 72.4 ± 2.73 ng/mL vs. 53.9 ± 2.99 ng/mL; *P* = 0.038). No interaction effects with breed on 25(OH)D concentrations were found. A main effect of vaccination status on 25(OH)D concentrations was also observed [*F*(1, 19) = 5.02; *P* = 0.037]: the mean concentrations of the vaccinated group were higher than those of the unvaccinated group (42.8 ± 1.47 ng/mL vs. 37.8 ± 1.60 ng/mL). Time was found to affect 25(OH)D concentrations [*F*(15, 298) = 66.97; *P* < 0.001], with concentrations increasing with time up to wk 54 and flattening out subsequently (Figure 1). A main effect of breed on 25(OH)D concentrations was observed [F(1, 19) = 6.87; *P* = 0.017], with Holstein-crossbred cattle having higher 25(OH)D levels than purebred cattle (43.2 ± 1.47 ng/mL vs. 37.4 ± 1.62 ng/mL).

**Figure 1:**
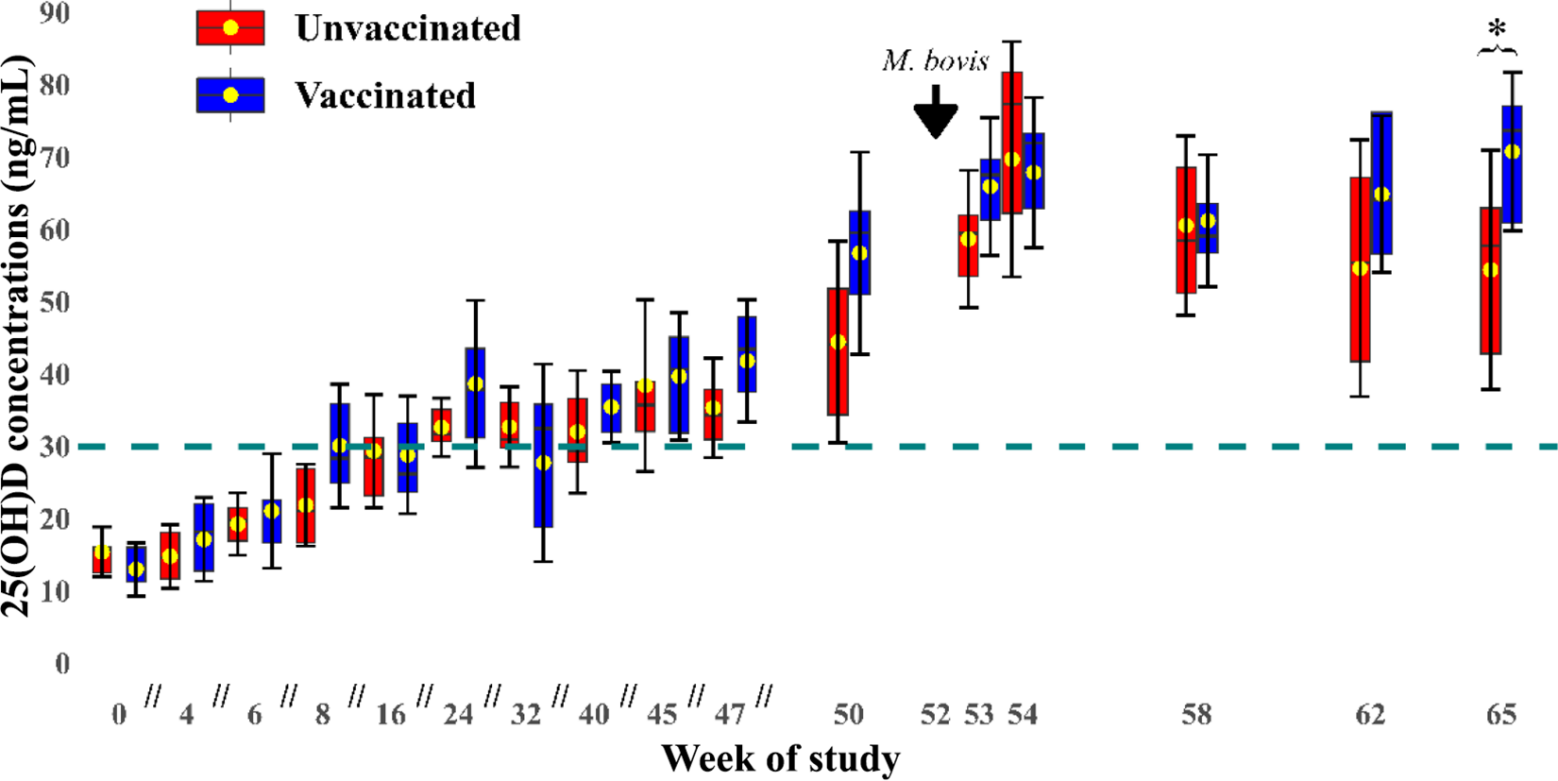
Boxplot of the relationship between 25(OH)D concentrations and time stratified by vaccination status (red bars: unvaccinated; blue bars: vaccinated calves). Vaccination with BCG took place on wk 0, whereas infection with *Mycobacterium bovis* happened on wk 52. Time points are indicated for *M. bovis* infection. The x-axis is discontinuous, with breaks marked by separators (//), with a transition to continuous from wk 50 onward. The central line represents the median, and the box spans the interquartile range. The mean is represented with a yellow point. Error bars (SD) are included for each mean. The horizontal dotted line represents the suggested vitamin D insufficiency reference point at 30 ng/mL. *Significant difference (*P* < 0.05).

A main effect of vaccination status on LN pathology scores was observed [*F*(1, 19) = 7.36; *P* = 0.014], with previously vaccinated calves having lower LN pathology scores than unvaccinated ones. An effect of 25(OH)D concentrations on LN pathology scores was found at wk 62 (10 wk pi) [*F*(1, 18) = 8.72; *P* = 0.009] followed by a tendency at wk 65 (13 wk pi) [*F*(1, 19) = 4.17; *P* = 0.055] (Figures 2A and 2B). The vaccination status affected the intercept of the linear relationship (*P* = 0.022) but did not affect the slope of the relationship. Vaccination status did not affect the slope or the intercept of the relationship at wk 65 (13 wk pi).

**Figure 2:**
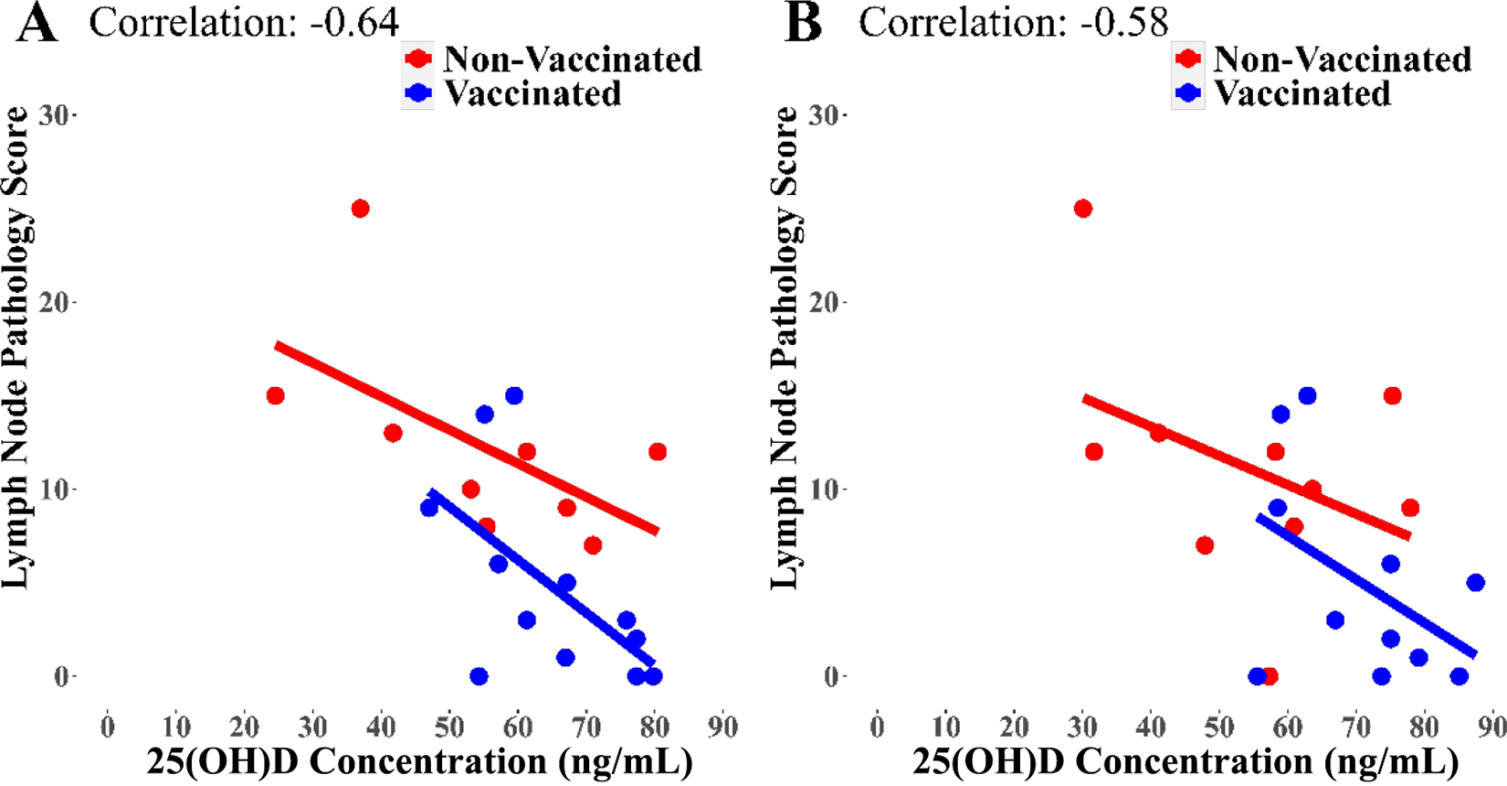
Scatter plot of the relationship between 25(OH)D concentrations and the LN pathology score for *Mycobacterium bovis* infection between the vaccinated and unvaccinated animals on wk 62 (10 wk pi; A) and wk 65 (13 wk pi; B). In both cases, the relationship was linear. Vaccination status affected only its intercept on wk 62 but did not affect the intercept or the slope of the relationship at wk 65.

A main effect of vaccination status on lung pathology scores was found [*F*(1, 19) = 6.87; *P* = 0.017], with previously vaccinated calves having lower pathology scores than unvaccinated ones. Concentrations of 25(OH)D tended to affect lung pathology scores at wk 65 (13 wk pi) [*F*(1, 19) = 3.47; *P* = 0.078]. The vaccination status affected only the intercept of the linear relationship at wk 62 (10 wk pi; *P* = 0.009). Vaccination status did not affect the slope or the intercept of the relationship at wk 65 (13 wk pi).

Similar effects of vaccination status and 25(OH)D concentrations on total (LN plus lung) pathology scores were also identified. Concentrations of 25(OH)D tended to affect total pathology scores at wk 62 (10 wk pi) [*F*(1, 19) = 3.01; *P* = 0.099], and had an effect on total pathology scores at wk 65 (13 wk pi) [*F*(1, 18) = 4.64; *P* = 0.044]. The vaccination status affected the intercept of the linear relationship only at wk 62 (10 wk pi; *P* = 0.011).

The partial correlation coefficient for the relationship between 25(OH)D concentrations and IFN-γ, while controlling for vaccination status and time, was −0.066 (*P* = 0.499). This is consistent with findings from Ragab et al. (2016); however, others have reported a correlation between higher vitD levels and reduced IFN-γ production in vitro (Barker et al., 2013).

We accept there may be some limitations in this study (e.g., the absence of food intake data), as investigating vitD was an opportunist additional piece of work in a TB vaccination-protection investigation. Nevertheless, efforts were made to ensure uniform treatment of all animals regarding accommodation, feed, and exposure to light sources.

Consistent with the first hypothesis, there was no difference in 25(OH)D concentrations between the vaccinated and unvaccinated calves pre-infection. We found no differences in BW between the vaccinated and unvaccinated animals, suggesting that the vaccination did not significantly affect feed intake, as all animals were offered a BW-based allowance (Golder et al., 2021). Similar studies with BCG vaccination in cattle report that the animals did not show adverse effects (Balseiro et al., 2020). However, Williams et al. (2022) reported inappetence in calves as a transient factor only in the first 14 d postvaccination. Differences in baseline 25(OH)D levels between the purebred Holstein and the crosses were also identified, emphasizing the importance of accounting for breed-related variations in vitD research in cattle. We also note that all animals had 25(OH)D concentrations lower than the suggested vitD insufficiency threshold of 30 ng/mL until experimental wk 6 (∼3 mo of age), and many were still below this level up to wk 16 (∼5 mo of age).

Regarding the second hypothesis of the study—that *M. bovis* infection would result in differences in 25(OH)D between vaccinated and unvaccinated cattle, in keeping with a role for vitD in vaccine-induced protection—BCG-vaccinated calves did show a divergence in 25(OH)D levels compared with unvaccinated cattle pi. A transient drop in mean 25(OH)D was observed 6 wk pi (wk 58) in both groups—potentially associated with a transient reduction in food intake (pathogen-induced anorexia) that is consequently reflected in their circulating 25(OH)D concentrations (Aşkar et al., 2018). Unfortunately, BW was not recorded pi to confirm.

Previous literature reports that the lag time between the point of infection and the first signs of anorexia for bacterial infections can vary based on the time course of recognition of the pathogen by host immunity (Kyriazakis et al., 1998). The temporal developments in 25(OH)D measurement support a potential faster rate of recovery from pathogen-induced anorexia in vaccinated animals compared with unvaccinated calves (Sandberg et al., 2007). Vaccinated calves, having acquired immunity to the *M. bovis* pathogen, might exhibit a quicker and more effective immune response, possibly resulting in a shorter anorexia duration, if any (Plata-Salamán, 2001). However, the statistically significant differences in 25(OH)D concentrations were observed at wk 65 only, and caution should be exercised in drawing generalized conclusions.

The third hypothesis of the study—that serum 25(OH)D levels would associate with the level of pathology—was upheld by the significant (*P* = 0.017) negative correlation of higher pathology score with lower 25(OH)D concentrations. This was the case in lung, LN, and total pathology scores, linking higher 25(OH)D levels with effective immunity (less pathology) (Hope et al., 2023).

Although there is a lack of research in cattle, there are proposed mechanisms in other species which could potentially explain the effects of 25(OH)D concentrations on the expression of immunity in infected hosts (Thirumdas et al., 2021). The inflammatory response and granuloma formation is essential in the early stages of TB disease; however, persistent granulomatous inflammation is the main cause of tissue damage and clinical manifestation of the disease (Sasindran and Torrelles, 2011). It has been demonstrated that TB infection becomes uncontrolled whereby granuloma formation migrates away from the infection site to other tissues, causing subsequent damage (Capuano et al., 2003). Vitamin D has been identified within such granulomas in *M. bovis*-infected cattle (Rhodes et al., 2003). Vitamin D has demonstrated immunomodulatory effects, including the induction of T-reg lymphocytes, which limit Th1 activity via modulation of cytokine expression without the reduction of antimycobacterial activity, and the modification of co-stimulatory molecules on accessory cells (Rhodes et al., 2003; Fabri et al., 2011). This potentially explains how 25(OH)D concentrations may contribute to reduction of pathology, as the reduction of the inflammatory state will in turn reduce damage to the LN and lung tissues pi. Increased local production of 25(OH)D by these immune cells during infection could also potentially influence dendritic cell migration to the LN whereby further 25(OH)D production could continue, which would explain why the effect of 25(OH)D on pathology score becomes significant in the LN before the lungs (Sigmundsdottir and Butcher, 2008).

Although this study contributes to our understanding of the complex interactions between vaccination, infection, and vitD status in cattle, suggesting a role for vitD in the outcomes of TB infection, further research is essential to elucidate the underlying mechanisms responsible. Specifically, investigations into the pathways involved in the interplay between vaccination efficacy, infection susceptibility, and vitD modulation are warranted. The findings from such studies could have important implications (e.g., in the use of vitD supplementation to improve TB vaccination or control strategies). Furthermore, understanding the role of vitD in modulating immune responses could facilitate the development of novel therapeutic interventions.
